# The Hoof Color of Australian White Sheep Is Associated with Genetic Variation of the *MITF* Gene

**DOI:** 10.3390/ani13203218

**Published:** 2023-10-15

**Authors:** Peng Su, Hui Wu, Yangming Huang, Xiaofang Lu, Jing Yin, Qingfeng Zhang, Xianyong Lan

**Affiliations:** 1 Tianjin Aoqun Animal Husbandry Co., Ltd., Tianjin 301607, China; supersu@nwafu.edu.cn (P.S.);; 2 Key Laboratory of Animal Genetics Breeding and Reproduction of Shanxi Province, College Animal Science and Technology, Northwest A&F University, Yangling 712100, China; 3 National Germplasm Center of Domestic Animal Resources, Institute of Animal Science, Chinese Academy of Agricultural Sciences (CAAS), Beijing 100193, China; 4 College of Animal Science and Technology, China Agricultural University, Beijing 100193, China; 5 Tianjin Aoqun Sheep Industry Academy Company, Tianjin 301607, China

**Keywords:** sheep, GWAS, SNP, *MITF* gene, hoof color

## Abstract

**Simple Summary:**

In this research, we used RAD-seq genotyping data and GWAS to identify SNPs in the *MITF* gene that were significantly associated with hoof color in the Australian White (AUW) sheep. The results showed that the three genotypes of this SNP were linked to black, grey, and amber hoof color. These findings provide molecular markers that can be used in marker-assisted selection to select for hoof color of purebred AUW sheep.

**Abstract:**

Studying the characteristics of mammalian hoof colors is important for genetic improvements in animals. A deeper black hoof color is the standard for breeding purebred Australian White (AUW) sheep and this phenotype could be used as a phenotypic marker of purebred animals. We conducted a genome-wide association study (GWAS) analysis using restriction site associated DNA sequencing (RAD-seq) data from 577 Australian White sheep (black hoof color = 283, grey hoof color = 106, amber hoof color = 186) and performed association analysis utilizing the mixed linear model in EMMAX. The results of GWAS demonstrated that a specific single-nucleotide polymorphism (SNP; g. 33097911G>A) in intron 14 of the microphthalmia-associated transcription factor (*MITF*) gene was significantly associated with the hoof color in AUW sheep (*p* = 9.40 × 10^−36^). The *MITF* gene plays a key role in the development, differentiation, and functional regulation of melanocytes. Furthermore, the association between this locus and hoof color was validated in a cohort of 212 individuals (black hoof color = 122, grey hoof color = 38, amber hoof color = 52). The results indicated that the hoof color of AUW sheep with GG, AG, and AA genotypes tended to be black, grey, and amber, respectively. This study provided novel insights into hoof color genetics in AUW sheep, enhancing our comprehension of the genetic mechanisms underlying the diverse range of hoof colors. Our results agree with previous studies and provide molecular markers for marker-assisted selection for hoof color in sheep.

## 1. Introduction

Different mammal species exhibit striking and varied coat colors, which are a frequent and obvious biological occurrence [1]. This variation in coat colors, also known as coat-color polymorphism, is frequently observed between and within different species [2]. The diversity in coat colors among mammals is primarily a result of natural selection. The coat color of a particular taxon frequently reflects its adaptation to its environment, including its ability to blend in with its surroundings, capture prey, evade predators, attract mates, and protect against harmful ultraviolet rays [3]. In addition to the role of natural selection, the coat color of animals has also been subject to strong artificial selection by humans because it is a significant economic trait in many domesticated animals [4,5,6]. To investigate and understand the formation mechanism of different coat colors in mammals is an important area of research in genetics and evolutionary biology. The synthesis of coat color pigments is regulated by a complex network of genetic and environmental factors, and variations in these factors can lead to variations in the coat color [7]. Melanin types can be classified into two categories: eumelanin, which is a true melanin that does not contain sulfur atoms, is insoluble in various solvents, and primarily appears in shades of brown and black; and pheomelanin, which contains sulfur atoms, is a spherical red granule that is soluble in alkali and appears in shades of yellow or reddish-brown. The ratio of eumelanin to pheomelanin in an individual can result in different coat colors [8]. Several studies have shown that the formation of melanin is divided into four main stages: first, the formation of melanosomes; second, the maturation of melanosomes; third, the synthesis of melanin; and finally, the transport of melanosomes containing large amounts of melanin [9]. Melanin plays a key role in the formation of mammalian hair color. Its formation is regulated by a complex mechanism that involves multiple genes, from melanocyte differentiation and maturation to melanin synthesis and transport [10]. An increasing number of studies have shown that microRNAs (miRNAs) can also regulate melanin deposition, thus affecting the color of mammalian coats [11,12,13]. The coat color is an important economic trait in sheep for wool and skin. The mining of the candidate gene loci affecting the formation of different coat colors provides a reference for the genetic mechanism and convergent evolution of coat-color diversity in sheep [14]. While direct studies on hoof color of sheep remain sparse, research in related areas, such as coat color offers valuable insights, as these color characteristics are predominantly regulated by genes associated with pigment deposition.

The Australian White (AUW) breed of sheep originated in Australia. It is a coarse-wool and specialized mutton breed. It integrates the genes of four breeds of sheep; namely, Dorper, Van Rooy, Polled Dorset, and Texel. The AUW breed has the advantages of strong stress resistance, a fast growth rate, and easy management. It is used for both meat and wool production. This indicates that AUW sheep are suitable for crossbreeding with local sheep breeds, thereby improving the meat quality and production efficiency. In the case of AUW sheep, the hoof color can vary depending on the strain or pedigree. The hoof color can be black, grey, amber, or striped due to a combination of different pigments (Australian White Sheep Breed Standards, 2015). A deeper black hoof color is the standard for breeding purebred AUW sheep. Animal color traits, including hoof color, are qualitative traits controlled by a few genetic motifs. The color primarily depends on the type of melanin and its relative amount [15].

Since 2005, following the publication of the first GWAS paper on human macular degeneration, an increasing number of researchers have used GWAS to investigate various complex human diseases [16]. GWAS studies have made significant progress in understanding human diseases, notably those related to height [17], cancer [18], and blood pressure [19]. In recent years, GWAS studies have also identified numerous variant loci influencing crucial economic traits in animals [20,21,22]. Since the process of domestication, livestock have exhibited a diverse range of phenotypes, encompassing variations in coat color. Some research has indicated the presence of pleiotropy associated with certain genes that influence coat color, with some mutations potentially leading to lethal diseases [23]. Coat colors associated with recessive genetic diseases often lead to fatal consequences. This not only affects the survival and health of the animals but also results in significant economic losses [24]. The identification of these mutations provides a reliable reference for future Marker-Assisted Selection (MAS). In the past five years, the FAANG (Functional Annotation of Animal Genomes) project [25] and FARM-GTEx (Farm animals gene tissue expression) project have gained significant attention, particularly in the context of livestock such as pigs, chickens, cattle, and sheep [26]. These initiatives aim to establish comprehensive gene expression atlases to elucidate the variations in key genes across different tissues and physiological states [27]. By selecting AUW sheep with black hoof color, we aim to choose more purebred AUW sheep, thereby eliminating individuals that do not meet the breed standards. These efforts play a crucial role in advancing animal genomics research, ultimately contributing to improvements in the livestock industry and enhancing our understanding of the functional genomics of animals [28].

In this study, we sequenced the genomes of AUW sheep by RAD-seq. We aimed to excavate genes and loci influencing hoof color in AUW sheep according to the GWAS analysis. The GWAS results indicate that the highest signal was located within the *MITF* gene.

## 2. Materials and Methods

### 2.1. Animals and Hoof Color Data Collection

**Figure 1:**
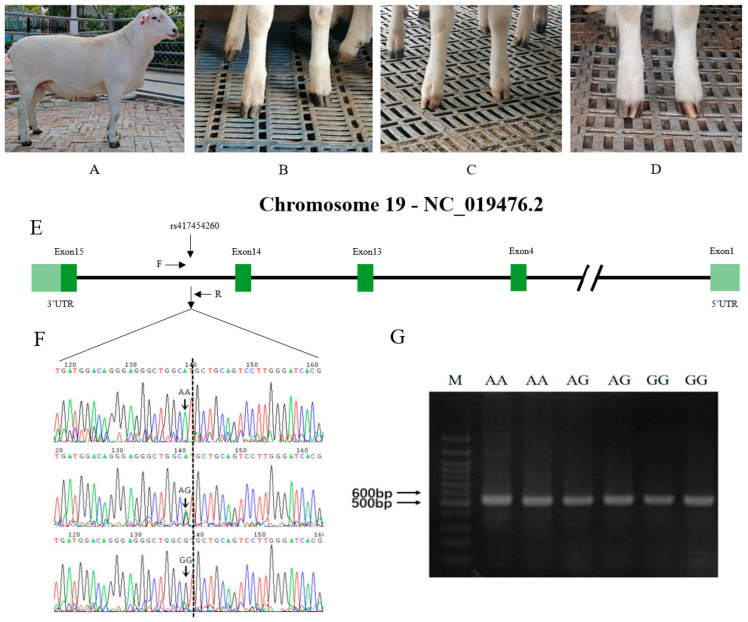
Agarose electrophoresis and sequencing chromas of SNP (g.31603203) in sheep *MITF* gene. (**A**) The Australian White sheep; (**B**) AUW sheep with black hoof color; (**C**) AUW sheep with grey hoof color; (**D**) AUW sheep with amber hoof color; (**E**) The position and gene structure of *MITF* gene in sheep; (**F**,**G**) The electrophoresis pattern and sequence chromatogram of the SNP (g.31603203) in AUW sheep *MITF* gene.

The AUW sheep analyzed in this study were reared on the sheep farm of the Aoqun Animal Husbandry Co., Ltd., Tianjin, China. A total of 789 AUW sheep were randomly selected from this farm. The sheep population consisted of 486 rams and 303 ewes. The hoof colors of the rams and ewes included in this study are presented in Table 1. All selected AUW sheep were approximately 1–2 years old, were healthy, and had been reared on the same farm with the same breeding environment, the same diet, and the same environmental conditions. Using the sheep earmark number for identification, the hoof color information was collected from the network database of Aoqun Animal Husbandry Co., Ltd. According to a hoof color inspection, the hoof colors of all the sheep were divided into three types: amber, grey, and black. We used 577 AUW sheep for the GWAS analysis and used the remaining 212 AUW sheep for the validation experiments. These were including black, grey, and amber hoof colors (Figure 1). The hoof colors of the GWAS and validation group included in this study are presented in Table 2 and Table 3. We collected the phenotypic data of the three hoof colors of the AUW sheep from the earmark numbers in the online database of Aoqun Animal Husbandry Co., Ltd.

### 2.2. Whole Genome Sequencing, Genotyping and Quality Control 

We collected the ear tissues from 789 AUW sheep for genomic DNA extraction. We used the high-salt extraction method to extract the DNA [29]. Sequencing of 577 AUW sheep was assigned to BGI Co., Ltd. (Shenzhen, China) for restriction site-associated DNA sequencing (RAD-seq). Double-end sequencing (PE150) of genomic DNA extracted from sheep ear tissue samples was performed using the Hiseq × ten sequencing platform and RAD-seq. 

After obtaining sequencing data, individual data were extracted based on tag sequences, low-quality sequencing fragments were filtered out, and high-quality fragments were aligned to the sheep reference genome (Oar_v4.0, https://www.ncbi.nlm.nih.gov/genome/?term=sheep, (accessed on 10 January 2023)) using BWA (version 0.7.17) software. We used Samtools (version 1.7) to sort the bam files and remove duplicates (-sort; -rmdup). The next step was to call SNPs by the GATK (version 4.1.9.0) software (-HaplotypeCaller) [30].

We used Vcftools (version 0.1.13) to control the quality of the raw data. The initial SNP data were quality controlled with the following criteria: call rate > 0.7, SNP quality > 20, minor allele frequency > 0.05, heterozygosity < 0.9, and HWE > 1 × 10^−6^. The fastPHASE software (version 1.4) was used to fill in the missing genotypes, and the filled SNPs were filtered again using the same quality control standards [31]. We used PLINK (version 1.90) to perform PCA (Principal Component Analysis, -pca 3) based on the genotypic data generated for AUW sheep.

### 2.3. GWAS of Hoof Color Traits in Australian White Sheep

We performed GWAS analysis using the genotypes resulting from the variant calling analysis previously performed for the RAD-seq data of 577 AUW sheep. GWAS analysis of the hoof color was performed using the EMMAX program (http://genetics.cs.ucla.edu/emmax/index.html, accessed on 10 January 2023). The mixed linear model (MLM) was used as follows: *y* = *Xb* + *Zu* + *m* + *e.*

In the model, *y* denotes hoof color, *X* denotes fixed effects association matrix, *b* denotes fixed effects vector, fixed effects include year-season, number of littermates, sex, and three principal component effects, *Z* denotes additive genetic effects association matrix, *u* denotes individual additive genetic effects vector, *e* denotes residuals, $u\sim N\left(0,G\sigma_{a}^{2}\right)$, $e\sim N\left(0,I\sigma_{e}^{2}\right)$, *G* denotes the genomic association matrix, *I* denotes the identity matrix, $\sigma_{a}^{2}$, $\sigma_{e}^{2}$, denotes additive genetic effects variance and residuals variance, respectively, and m denotes SNP marker effects. In this GWAS model, all available SNPs were included in the analysis. *p*-values were used to apply a Bonferroni correction and define the genome-wide (0.05/n, n = SNP numbers) and chromosome-wide (1/n, n = number of SNPs) significance thresholds [32].

### 2.4. Linkage Disequilibrium and Haplotype Analysis

The SNPs within a 200 Kb region centered around the top SNP identified by the GWAS were extracted by Bcftools (version 1.8). Subsequently, the linkage disequilibrium between these SNPs was calculated. We used LDblockshow (version 1.40) for data visualization. Annotation of SNP loci was performed using Variant Effect Predictor (VEP) software of the Ensembl database (https://asia.ensembl.org/info/docs/tools/vep/index.html, accessed on 5 June 2023).

### 2.5. Validation of Selected SNP in Six Australian White Sheep

We selected ear tissue DNA samples from six of the 789 AUW sheep based on the phenotypic data of hoof color, two for each of the three hoof colors.

We used the NCBI Primer Blast online analysis software (https://www.ncbi.nlm.nih.gov/tools/primer-blast/index.cgi, accessed on 24 March 2023) to design the primers of PCR amplification. We used the touchdown-PCR to amplify the target DNA fragment. We used a 50 µL system for amplification which included 2.5 μL of DNA template, 1.25 μL of each primer, 25 μL of PCR Mix reagent, and 20 μL of double-distilled water [33]. 

The PCR product was detected using 1.5% agarose gel electrophoresis. Sanger Sequencing was performed by the Tsingke Biotechnology Company (Xi’an, China) to verify mutations. The sequencing results were compared and analyzed with the sequence of the relevant gene fragment of sheep in GenBank using SnapGene software (https://snapgene.cn/, accessed on 24 March 2023). 

### 2.6. Association between the Hoof Color and Different SNP Genotypes in a Validation Population of 212 AUW Sheep

An association analysis was performed using the phenotypic data of the hoof color of 212 AUW sheep with Sanger sequencing data. The association analysis of the SNP in the *MITF* gene with the hoof color trait was performed using SPSS (Version 25.0, International Business Machines Corporation, New York, NY, USA). We used Chi-square tests to analyze the relationship between the variant in the *MITF* gene and the hoof color of AUW sheep. The linear model used was: Yij = µ + Si + Gi + eij. (Yij: the phenotypic value of hoof color; µ: the overall average; Si: the fixed effect of sex; Gi: the fixed effect of the genotype; eij: random error). All results were considered significant when *p* < 0.05. All tests of significance were two-sided tests.

### 2.7. Bioinformatics Analysis

We downloaded amino acid sequences of the *MITF* gene from six different species (*Ovis aries* (XP_004018379.2), *Capra hircus* (XP_013829095.2), *Bos taurus* (XP_024838185.1), *Homo sapiens* (NP_001341533.1), *Mus musculus* (NP_001106669.1) and *Sus scrofa* (XP_020924242.1)) in the NCBI database. The similarity of the protein sequences was calculated using Protein Blast (https://blast.ncbi.nlm.nih.gov/Blast.cgi, accessed on 30 March 2023). We used MEGA (Version 6.06) to align the multiple protein sequences and construct the phylogenetic tree [34]. 

In this study, we used relevant data from the Ruminant Genome Database of Animal Omics Database to explore the expression of the *MITF* gene in different tissues and KEGG (Kyoto Encyclopedia of Genes and Genomes) pathways (https://animal.nwsuaf.edu.cn/, accessed on 30 March 2023) [35]. The protein interaction network of MITF protein was analyzed by String online analysis software (https://string-db.org/, accessed on 30 March 2023). Genomic regions and candidate genes were recognized by a genome browser (NCBI). The corresponding SNP loci were obtained by GWAS. 

## 3. Results

### 3.1. Descriptive Statistics

The alignment was conducted using the BWA software, followed by data sorting and duplicate removal using Samtools. SNP calling was performed using GATK, resulting in a total of 175,902 SNPs. The fastPHASE software was used to impute the missing genotypes, and the imputed SNPs were filtered again using the same quality control standards. After quality control, a total of 577 AUW sheep and 171,842 SNPs were used in the GWAS analysis. The filtered SNPs were spread across all 26 chromosomes. Chromosome 1 had the highest number of SNPs and chromosome 24 had the lowest. The minor allele frequency (MAF) for all SNPs was recalculated following the quality control process, and only SNPs with an MAF of 5% or greater were retained.

### 3.2. Genome-Wide Association Study

**Figure 2:**
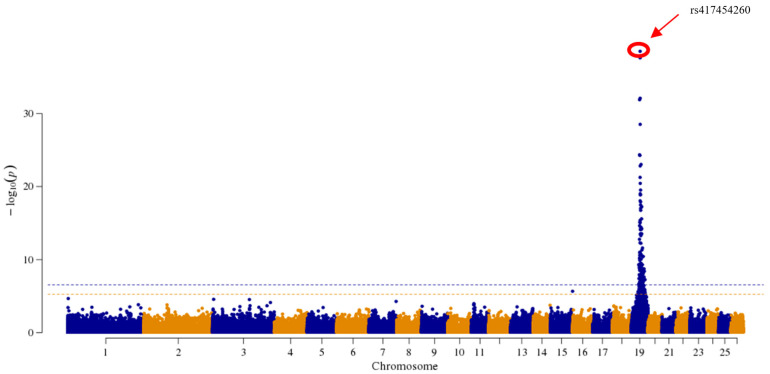
Manhattan plots of the hoof color of AUW sheep. *p*-value of each SNP and the location of the gene closest to each significant SNP of hoof color in the Manhattan plots.

**Figure 3:**
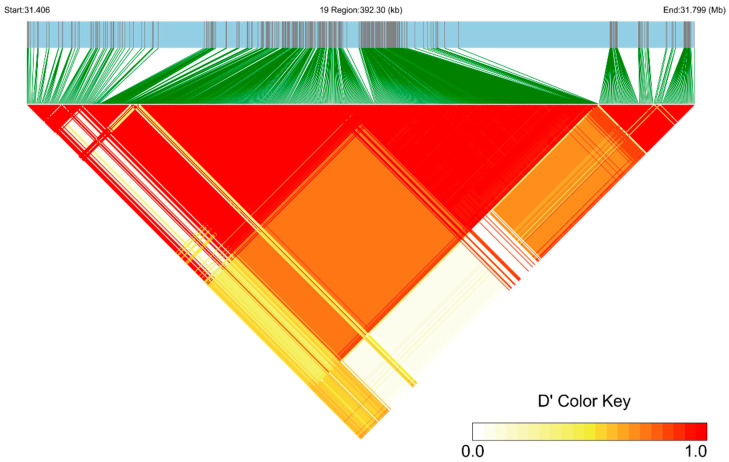
Linkage disequilibrium (LD) analysis spanning the physical position from 34.406 to 31.799 Mb of chromosome 19. The color key indicates the level of LD (r^2^) between variants in AUW sheep.

In this study, GWAS analysis was performed using the MLM previously described with the EMMAX software. After Bonferroni correction, the genome-wide significance threshold was 2.910 × 10^−7^, and the chromosome-wide significant threshold was 5.819 × 10^−6^. The accuracy and reliability of the results were improved by incorporating factors (year-season, number of littermates, sex, and three principal component effects) in the model. The top SNP locus was mined by GWAS analysis (*p* = 9.40 × 10^−36^) (Figure 2). This SNP was found at position 31,603,203 bp on chromosome 19 of the Oar_v4.0 reference sequence, and it has an A/G polymorphism. This SNP is situated in the intron 14 region of the *MITF* gene, which is associated with melanogenesis. We used the LDBlockShow to perform an LD analysis around the top SNP locus. The region of 200 kb before and after this SNP locus was at a high level of linkage disequilibrium (Figure 3). We found that most of the SNPs of this region are at high levels of linkage. Within this region, we identified a total of 561 SNPs, and analysis based on the VEP software showed that most SNPs are located in introns and intergenic regions, with only a few SNPs causing synonymous mutations and several SNPs situated within alternative splicing regions.

### 3.3. Validation of SNP Loci

The primers were designed using NCBI online software. The forward primer was GGCCACCTGATGTGAAGAAC and the reverse primer was TTGACAGTGTTGTGGGGCA (Table 4). The PCR product was visualized using electrophoresis on a 1.5% agarose gel. The size of the amplified target fragment was 529 bp. The genotype of these sheep was confirmed by Sanger sequencing (Figure 1). The sequence was consistent with the sequence information described in the NCBI database. The SNP site at g.31603203 A/G was identified at the 173rd position of the sequence. It was observed that individuals with the AA genotype showed a tendency towards amber hoof color, those with the AG genotype showed a tendency towards grey hoof color, while GG genotype showed a tendency towards black hoof color.

### 3.4. Association Analysis of the Locus in the MITF Gene

**Figure 4:**
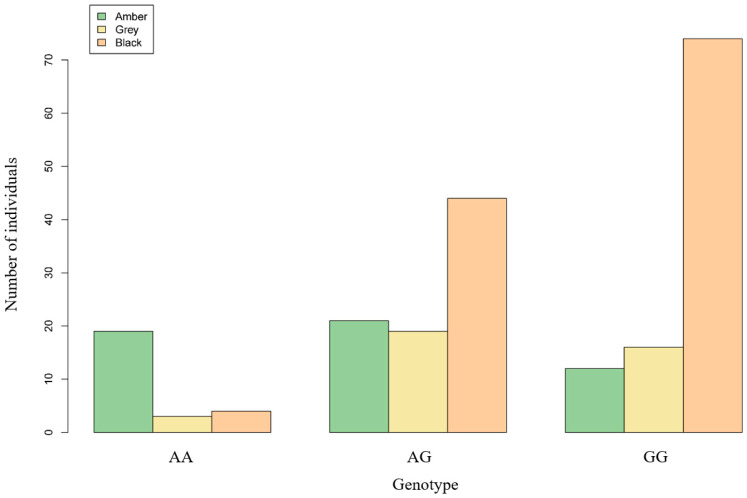
Three different genotypes of 212 sheep with different hoof colors.

A validation flock of 212 AUW sheep was obtained from the population of 577 sheep genotyped by Sanger sequencing and recorded by hoof color, but not included in the GWAS. As shown in Table 5, the frequency of G alleles at locus g.31603203 A/G was highly significantly associated with the hoof color (*p* < 0.01). The more G alleles an individual had, the darker the hoof color (Figure 4).

### 3.5. Construction of Phylogenetic Tree of MITF Gene in Six Species and Gene Expression

**Figure 5:**
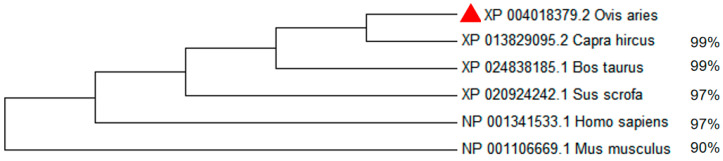
Phylogenetic tree based on the amino acid sequences of *MITF* gene among different species. Note: The red triangle represents the sheep species in this study.

**Figure 6:**
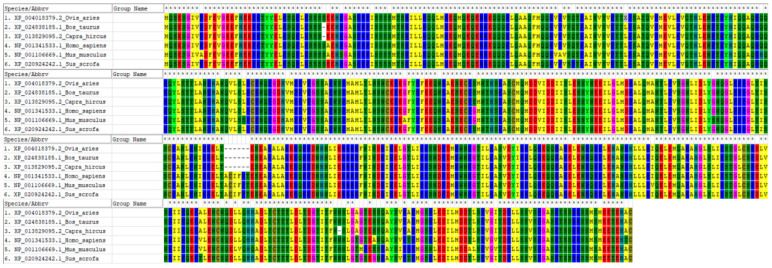
Amino acid sequence alignments of *MITF*. Identical residues aligned and colored by the MEGA6 tool are indicated under each column according to the consensus character assigned. The * indicates the core regions are of high similarity.

The *MITF* amino acid sequences of six species including *Ovis aries*, *Capra hircus*, *Bos taurus*, *Homo sapiens*, *Mus musculus*, and *Sus scrofa* were downloaded from the NCBI database. The NCBI BLAST analysis revealed that the ovine *MITF* gene exhibited high levels of identity with the *MITF* gene sequences from other species, as indicated by the percentages of 99%, 99%, 97%, 97%, and 90% (Figure 5). Sequence alignment of amino acids and phylogenetic tree results show that the *MITF* gene is highly conserved across multiple species and that the gene may have played an important role in the evolutionary process (Figure 5 and Figure 6).

## 4. Discussion

As animal breed characteristics or protective colors, color traits are often used in breed identification and species evolution, and are important phenotypes in the field of genetic breeding [36]. Several studies have concentrated on the coat color of animals [37,38,39,40,41]. Previous research reported that the alleles of the *MC1R* gene (s26449) were associated with pigmentation in sheep. This SNP (s26449) was located near the *MC1R* gene; mutations in this SNP were significantly associated with the coat colors of sheep [42]. As early as 2009, a study reported that a QTL on cattle chromosome 15 was significantly associated with hoof pigmentation. Two QTL regions in chromosome 22 and chromosome 6 were observed to be significantly associated with sole pigmentation in cattle. The *MITF* gene is located in the QTL region of chromosome 22 which could affect the spotting traits [43]. Researchers have reviewed the genes and variations that affect the coat color phenotype of domestic dogs. A few of these genes involved in canine pigmentation were also associated with hearing, visual, and nervous-system damage [15]. Using whole genome and transcriptome sequencing, the results demonstrated that the insertion of a 2809 bp long LINE-1 in the *ASIP* gene could lead to the white-hair phenotype of swamp buffalo [44]. The whole genome-association mapping of 16 captive tigers was performed using restriction endonuclease-related DNA sequencing (RAD-seq); whole genome sequencing (WGS) was then performed on the three parents. The causative mutation was observed to result in an amino acid change (A477V) in the transporter protein SLC45A2 after being validated in 130 unrelated tigers, which led to the formation of a white coat color [45]. Based on the previous studies, our work investigated the gene that affected the hoof color of AUW sheep and identified the SNP loci that significantly affect hoof colors using a GWAS analysis. Therefore, we conducted a GWAS analysis on the hoof colors of the AUW sheep population. We discovered a variant (rs417454260) in the *MITF* gene that was associated with hoof colors, including amber, grey, and black.

Melanin is a protein byproduct produced by melanosomes in melanocytes that is not soluble in water or most organic solvents. The ratio of true to brown melanin is strongly correlated with the activity of the enzyme tyrosinase (*TYR*). The *MITF* gene, a transcription factor involved in melanogenesis, codes for the MITF protein, which regulates the tyrosine gene family (*TYR*, *TYRP1*, and *TYRP2*) [46]. The MITF protein belongs to the TFE family of transcription factors and can bind to the CANNTG core sequence within the E-box or M-box in a dimeric form to control the transcriptional expression of its target genes [47]. *MITF* is a member of the CANNTG superfamily. Previous research has shown that the *MITF* gene plays a role in the development, differentiation, and functional regulation of melanocytes [48], uveal epithelial cells [49], and osteoblasts [50]. As a result, the *MITF* gene plays a critical role in the formation of true melanin and indirectly affects color traits in animals. Coat colors in sheep and other mammalian species can display various shades. The *MITF* gene is known to play a crucial role in the process of melanin deposition, which significantly contributes to the observed coat color. The promoter of the *MITF* gene, located directly upstream of the melanocyte isoform, is regulated by various transcription factors and signaling pathways that are involved in melanocyte and melanoma biology. *MITF* is a gene of significant interest that exerts pleiotropic effects on various fundamental cellular processes such as cell survival, invasion, senescence, differentiation, metabolism, proliferation, and DNA damage repair [47]. As the *MITF* gene is conserved, it plays a key role in pigmentation in other species. A 10 bp insertion in the melanocyte-specific promoter of the *MTIF* gene could explain a proportion of horses with white-spotting phenotypes [51]. Few studies have focused on the hoof colors of sheep. Before the present study, there was a lack of information available regarding the genes responsible for determining the hoof colors of AUW sheep. In this study, we studied three hoof colors (black, grey, and amber) of AUW sheep (Figure 1). In the MLM, we included the year-season, number of littermates, sex, and three principal component effects. Through the GWAS analysis, we identified the top SNP (rs417454260), which was significantly associated (*p* = 9.40 × 10^−36^) with the hoof colors of AUW sheep. We observed that this SNP locus was located in intron 14 of the *MITF* gene using gene annotation. 

In this study, we employed a combination of bioinformatics software analysis and experimental verification. We selected six additional sheep from the AUW sheep breed for validation purposes. The PCR products of these sheep were analyzed via agarose gel electrophoresis and were observed to have a size of 529 bp. The sequencing results were then compared and analyzed with the sequence of the relevant gene fragment of sheep from GenBank using SnapGene software. These results ensure the accuracy of subsequent Sanger sequencing. We associated the sequencing data with the hoof colors of 212 additional sheep from the AUW population. Our analysis results indicate that sex is not significantly associated with hoof color in AUW sheep. The association analysis indicated that the number of G alleles at the g.31603203A/G locus was highly significantly associated with the black hoof color (*p* < 0.01). Our association experiments confirmed the results of the GWAS analysis, revealing a significant association between the SNP locus (g.31603203A/G) and the hoof color of AUW sheep. According to an LD analysis, a 200 kb region around this SNP locus was highly linked. This suggested that this region had received a high degree of selection and that changes in this region may have resulted in different hoof colors of AUW sheep. Related studies identified SNP loci within a region of the *RXFP2* gene on chromosome 10; this peak was significantly associated with the horn phenotype [52]. It was noted that a region with more than 20 SNPs was significantly associated with brachygnathia, cardiomegaly, and renal hypoplasia syndrome disease in a Merino sheep population [53]. The length of this region was greater than 1.1 MB. These studies also demonstrated that the SNP locus we studied that significantly affected the hoof color of AUW sheep might not be a single SNP locus, but could be a highly selected region. Using the Ruminant Genome Database (Sheep Variation Database), we found the MAF of this SNP is 0.2824. Based on this database, we estimated the allele frequencies of this SNP in the different sheep breeds across the world (Figure S1). According to this database, the European breeds predominantly exhibit A allele, while Asian breeds tend to have a higher frequency of G allele. For example, we found that the allelic frequency of European Mofflen sheep was 1.00, and the allelic frequency of Guide Black Fur sheep was 0.00. These data further corroborate our results. We postulate that this locus is under significant artificial selection pressure. Based on this analysis, it has been observed that the frequency of this SNP among different regions in sheep populations (East Asia = 0.167; Central and West Asia = 0.111; South and Southeast Asia = 0.333; Europe = 0.368; America = 0.071; Africa = 0.486). The distribution of the frequency of this SNP can reflect the artificial selective pressures that sheep populations may have experienced under different environmental conditions, as well as the genetic variations that arise during adaptation to specific environments. Understanding the frequency of the SNP among different populations can provide valuable information for breeding and genetic improvement, aiding in the selection of purebred individuals for breeding.

The hoof color of sheep may be a complex trait that is determined by multiple genes, pathways, or networks. The *MITF* gene has been identified as a key determinant of the color phenotype. An analysis of the data from the Ruminant Genome Database also revealed that the *MITF* gene is highly expressed in the skin, as shown in Figure S2. This finding indirectly supports the notion that the *MITF* gene plays a crucial role in the process of melanin deposition. An analysis using STRING v11 software revealed an interaction network centered on MITF proteins, including the MAPK family proteins MAPK3 and MAPK1 as well as TYRP1 (tyrosinase-related protein 1), all within two steps of this network. The proteins that interact with MITF may also influence the expression of melanogenesis. Melanogenesis is a complex process that involves the synthesis of melanin in melanocytes and its subsequent transport to keratinocytes. This process is regulated by multiple genes and signaling pathways. DNA methylation regulates the expression of key genes that influence the expression of melanin, such as tyrosinase (*TYR*), *TYRP1*, dopachrome tautomerase (*DCT*), and *MITF*. Potential DNA methylation sites have been identified in the genes associated with melanogenesis-related signaling pathways such as Wnt, PI3K/Akt/CREB, and MAPK [54]. The IFNG–STAT1 pathway regulated melanogenesis via the regulation of the post-translational processing and protein stability of *TYR* [55]. The expressions of *TYR* and *TYRP1* genes in the head feather barbules of males were higher than in females. These genes significantly regulated the expression of melanin, which affected the color of the head in the different sexes [56]. Liu et al. used the CRISPR–Cas9 gene editing technology to target *TYR* in white crucian carp (WCC) and its hybrid progeny (WR), derived from the cross of WCC and red crucian carp. Their study observed that the level of TYR protein was significantly reduced in the mutant WCC compared with the wild-type sibling control fish, resulting in varying degrees of melanin reduction in both the mutant WCC and mutant WR; the mutation efficiency ranged from 60% to 90%. A series of pivotal pigment synthesis genes, including *TYRP1*, *MITFA*, *MITFB*, *DCT*, and *SOX10*, were downregulated in the TYR–CRISPR WCC, ultimately resulting in a reduction in melanin synthesis [57]. With the continuous development of sequencing technology, several studies have shown that mutations in the intron may also regulate the expression of genes [58]. Mutations in non-coding positions have also been noted to affect phenotypic changes, especially in cis-regulatory regions. Epigenetics could explain this mechanism [59]. The results of several studies have shown that the SNPs in introns might change the alternative splicing mechanism [60]. Further in-depth studies are required to detect the mechanism of this SNP.

From the GWAS results, we were able to identify an SNP (rs417454260) located within the *MITF* gene that was associated with the hoof color trait in AUW sheep. Currently, the selection of the hoof colors of AUW sheep is mainly based on the subjective evaluation of hoof colors by human observers, which can lead to differences in interpretation and reduce the accuracy of the selection. In contrast, MAS uses the genetic information of the animals themselves, rather than a subjective evaluation to improve the accuracy of the selection. However, there is a lack of molecular markers that are available for hoof color selection. Identifying and screening the molecular markers related to hoof colors aids the accelerated breeding of AUW sheep strains with a consistent hoof color. This research may assist with the identification of valuable genes and mutations in the AUW sheep, improving the existing genetic resources and preparing for future large-scale meat-sheep breeding. Studying the genetic basis of the hoof color may lead to the discovery of genes related to pigment-associated diseases (such as albinism and melanoma), which could aid in the development of new treatment methods. Such studies could help in the understanding of the mechanisms of a range of human diseases caused by melanin deposition using sheep as an animal model. The hoof color serves as a tool to discern purebred AUW sheep populations. In this research, we discovered that the *MITF* gene is likely to affect the hoof color in AUW sheep. These findings contribute to a more comprehensive understanding of the genetic foundations underlying the hoof colors of sheep and affirm the association of this locus with the phenotype. These results can be applied in future MAS breeding strategies for purebred AUW sheep. They can also be used in sheep breeding, ultimately enhancing the economic efficiency of the sheep industry. Further research is required to refine these results and to expand upon the molecular mechanisms governing the inheritance of the hoof colors of sheep.

## 5. Conclusions

This study identified a SNP marker in the color-related *MITF* gene that is associated with hoof color in the AUW sheep breed. The most significant SNP identified, located in an intron of this gene (rs417454260), could be considered a candidate genetic marker in relation to hoof variation in AUW sheep. This study offers an opportunity for MAS breeding in the future. These findings provide molecular markers that can be used in marker-assisted selection to select and breed the hoof colors of purebred AUW sheep, contributing to a better understanding of the genetic basis of this trait.

## Figures and Tables

**Table 1:** Hoof Color Statistics for Total AUW Sheep.

Sex	Black	Grey	Amber
Ram	1.236	2.85	3.165
Ewe	4.169	5.59	6.75
Total	7.405	8.144	9.240

**Table 2:** Hoof Color Statistics of the Validation Group.

Sex	Black	Grey	Amber
Ram	78	22	33
Ewe	44	16	19
Total	122	38	52

**Table 3:** Hoof Color Statistics of the GWAS.

Sex	Black	Grey	Amber
Ram	158	63	132
Ewe	125	43	56
Total	283	106	186

**Table 4:** PCR Primer Sequences of the Sheep *MITF* Gene for Amplification.

Variant ID	Primer Sequences (5′ to 3′)	Location
rs417454260	F:GGCCACCTGATGTGAAGAAC	Intron 14
	R:TTGACAGTGTTGTGGGGCA	

**Table 5:** The Number of Hoof Colors for 212 AUW Sheep with Three Different Genotypes.

Genotype	Number of Individuals	Number of Individuals with Different Hoof Colors		
		Amber	Grey	Black
AA	26	19	3	4
AG	84	21	19	44
GG	102	12	16	74

## Data Availability

The datasets generated and analyzed during the current study are not publicly available, since the studied population consists of the nucleus herd of Tianjin Aoqun Sheep Industry Academy Co., Ltd., Tianjin, China, but are available from the corresponding author on reasonable request.

## Supplementary Materials

The following supporting information can be downloaded at: https://www.mdpi.com/article/10.3390/ani13203218/s1, Figure S1. Genotyping of the rs417454260 SNP in the global sheep population. These data were downloaded from the Ruminant Genome Database. Wild_Ref means wild sheep with the G allele; Wild_Alt means wild sheep with the A allele; Dom_Ref means domestic sheep with the G allele; Dom_Alt means domestic sheep with the A allele. Figure S2. The *MITF* gene expression in sheep tissues at adult stage. These data were downloaded from the Ruminant Genome Database.

## References

[1] Klungland H., Vage D. (2003). Pigmentary switches in domestic animal species. Ann. N. Y. Acad. Sci..

[2] Xu X., Dong G., Schmidt-Küntzel A., Zhang X., Zhuang Y., Fang R., Sun X., Hu X., Zhang T., Yang H. (2017). The genetics of tiger pelage color variations. Cell Res..

[3] Protas M., Patel N. (2008). Evolution of coloration patterns. Annu. Rev. Cell Dev. Biol..

[4] Royo L., Alvarez I., Arranz J., Fernández I., Rodríguez A., Pérez-Pardal L., Goyache F. (2008). Differences in the expression of the ASIP gene are involved in the recessive black coat colour pattern in sheep: Evidence from the rare Xalda sheep breed. Anim. Genet..

[5] Fontanesi L., Dall’Olio S., Beretti F., Portolano B., Russo V. (2011). Coat colours in the Massese sheep breed are associated with mutations in the agouti signalling protein (ASIP) and melanocortin 1 receptor (*MC1R*) genes. Animal.

[6] Cruz A., Yucra A., Gutiérrez G., Burgos A., Morante R., Gutiérrez J., Cervantes I., Wurzinger M. (2021). Colorimetry analysis of coat color and its relationship with fiber traits in alpacas. Animal.

[7] Chang T. (2009). An updated review of tyrosinase inhibitors. Int. J. Mol. Sci..

[8] Chen W., Tseng T., Hsiao N., Lin Y., Wen Z., Tsai C., Lee Y., Lin H., Tsai K. (2015). Discovery of highly potent tyrosinase inhibitor, T1, with significant anti-melanogenesis ability by zebrafish in vivo assay and computational molecular modeling. Sci. Rep..

[9] Wang L., Liu J. (2019). Research progress on molecular mechanism in the formation of melanin. J. Xinjiang Univ..

[10] Vandamme N., Berx G. (2019). From neural crest cells to melanocytes: Cellular plasticity during development and beyond. Cell Mol. Life Sci..

[11] Dong C., Wang H., Xue L., Dong Y., Yang L., Fan R., Yu X., Tian X., Ma S., Smith G. (2012). Coat color determination by miR-137 mediated down-regulation of microphthalmia-associated transcription factor in a mouse model. RNA.

[12] Fernández-Rodríguez A., Estellé J., Blin A., Muñoz M., Créchet F., Demenais F., Vincent-Naulleau S., Bourneuf E. (2014). KIT and melanoma predisposition in pigs: Sequence variants and association analysis. Anim. Genet..

[13] Kim K., Lee T., Cho E. (2017). SH3BP4, a novel pigmentation gene, is inversely regulated by miR-125b and MITF. Exp Mol. Med..

[14] Zhou Q., Cao C., Zhang H., Liang Y., Zhang X., Kang Y., Fang W., Lan X., Li R., Pan C. (2023). Convergent changes in melanocortin receptor 1 gene are associated with black-headed coat color in sheep. J. Anim. Sci..

[15] Brancalion L., Haase B., Wade C. (2022). Canine coat pigmentation genetics: A review. Anim. Genet..

[16] Klein R., Zeiss C., Chew E., Tsai J., Sackler R., Haynes C., Henning A., SanGiovanni J., Mane S., Mayne S. (2005). Complement factor H polymorphism in age-related macular degeneration. Science.

[17] Yengo L., Vedantam S., Marouli E., Sidorenko J., Bartell E., Sakaue S., Graff M., Eliasen A., Jiang Y., Raghavan S. (2022). A saturated map of common genetic variants associated with human height. Nature.

[18] Mavaddat N., Michailidou K., Dennis J., Lush M., Fachal L., Lee A., Tyrer J., Chen T., Wang Q., Bolla M. (2019). Polygenic Risk Scores for Prediction of Breast Cancer and Breast Cancer Subtypes. Am. J. Hum. Genet..

[19] Evangelou E., Warren H., Mosen-Ansorena D., Mifsud B., Pazoki R., Gao H., Ntritsos G., Dimou N., Cabrera C., Karaman I. (2018). Million Veteran Program. Genetic analysis of over 1 million people identifies 535 new loci associated with blood pressure traits. Nat. Genet..

[20] Li X., Yang J., Shen M., Xie X., Liu G., Xu Y., Lv F., Yang H., Yang Y., Liu C. (2020). Whole-genome resequencing of wild and domestic sheep identifies genes associated with morphological and agronomic traits. Nat. Commun..

[21] Liu X., Zhang Y., Liu W., Li Y., Pan J., Pu Y., Han J., Orlando L., Ma Y., Jiang L. (2022). A single-nucleotide mutation within the TBX3 enhancer increased body size in Chinese horses. Curr. Biol..

[22] Li Y., Gong Y., Zhang Z., Li L., Liu X., He X., Zhao Q., Pu Y., Ma Y., Jiang L. (2023). Whole-genome sequencing reveals selection signals among Chinese, Pakistani, and Nepalese goats. J. Genet. Genom..

[23] Voß K., Blaj I., Tetens J., Thaller G., Becker D. (2022). Roan coat color in livestock. Anim. Genet..

[24] Bellone R. (2010). Pleiotropic effects of pigmentation genes in horses. Anim. Genet..

[25] Andersson L., Archibald A., Bottema C., Brauning R., Burgess S., Burt D., Casas E., Cheng H., Clarke L., Couldrey C. (2015). FAANG Consortium. Coordinated international action to accelerate genome-to-phenome with FAANG, the Functional Annotation of Animal Genomes project. Genome Biol..

[26] Liu S., Gao Y., Canela-Xandri O., Wang S., Yu Y., Cai W., Li B., Xiang R., Chamberlain A., Pairo-Castineira E. (2022). A multi-tissue atlas of regulatory variants in cattle. Nat. Genet..

[27] Yao Y., Liu S., Xia C., Gao Y., Pan Z., Canela-Xandri O., Khamseh A., Rawlik K., Wang S., Li B. (2022). Comparative transcriptome in large-scale human and cattle populations. Genome Biol..

[28] Pan Z., Yao Y., Yin H., Cai Z., Wang Y., Bai L., Kern C., Halstead M., Chanthavixay G., Trakooljul N. (2021). Pig genome functional annotation enhances the biological interpretation of complex traits and human disease. Nat. Commun..

[29] Li H., Xu H., Akhatayeva Z., Liu H., Lin C., Han X., Lu X., Lan X., Zhang Q., Pan C. (2021). Novel indel variations of the sheep FecB gene and their effects on litter size. Gene.

[30] McCormick R., Truong S., Mullet J. (2015). RIG: Recalibration and interrelation of genomic sequence data with the GATK. G3 Genes Genomes Genet..

[31] Song S., Yao N., Yang M., Liu X., Dong K., Zhao Q., Pu Y., He X., Guan W., Yang N. (2016). Exome sequencing reveals genetic differentiation due to high-altitude adaptation in the Tibetan cashmere goat (*Capra hircus*). BMC Genom..

[32] Ghasemi M., Zamani P., Vatankhah M., Abdoli R. (2019). Genome-wide association study of birth weight in sheep. Animal.

[33] Su P., Luo Y., Huang Y., Akhatayeva Z., Xin D., Guo Z., Pan C., Zhang Q., Xu H., Lan X. (2022). Short variation of the sheep PDGFD gene is correlated with litter size. Gene.

[34] Zheng K., Su X., Zheng X., Zhang L., Chen Y., Wu K., Li T., Zhang Z., Zhao Z. (2021). First report of Stenotrophomonas maltophilia causing root soft rot of Sanqi (*Panax notoginseng*) in China. Plant Dis..

[35] Fu W., Wang R., Nanaei H., Wang J., Hu D., Jiang Y. (2022). RGD v2.0: A major update of the ruminant functional and evolutionary genomics database. Nucleic Acids Res..

[36] Fan Y., Wang P., Fu W., Dong T., Qi C., Liu L., Guo G., Li C., Ciu X., Zhang S. (2014). Genome-wide association study for pigmentation traits in Chinese Holstein population. Anim. Genet..

[37] Puckett E., Davis I., Harper D., Wakamatsu K., Battu G., Belant J., Beyer D., Carpenter C., Crupi A., Davidson M. (2023). Genetic architecture and evolution of color variation in American black bears. Curr. Biol..

[38] Leite J., Silva R., Asensio L., Sousa J., Silva W., Façanha D. (2020). Coat color and morphological hair traits influence on the mechanisms related to the heat tolerance in hair sheep. Int. J. Biometeorol..

[39] Wang C., Li H., Guo Y., Huang J., Sun Y., Min J., Wang J., Fang X., Zhao Z., Wang S. (2020). Donkey genomes provide new insights into domestication and selection for coat color. Nat. Commun..

[40] Muniz M., Caetano A., McManus C., Cavalcanti L., Façanha D., Leite J., Facò O., Paiva S. (2016). Application of genomic data to assist a community- based breeding program: A preliminary study of coat color genetics in Morada Nova sheep. Livest. Sci..

[41] Li M., Tiirikka T., Kantanen J. (2014). A genome-wide scan study identifies a single nucleotide substitution in ASIP associated with white versus non-white coat-colour variation in sheep (*Ovis aries*). Heredity.

[42] Kijas J., Serrano M., McCulloch R., Li Y., Ortiz J.S., Calvo J., Pérez-Guzmán M. (2013). International Sheep Genomics Consortium. Genomewide association for a dominant pigmentation gene in sheep. J. Anim. Breed Genet..

[43] Liu L., Harris B., Keehan M., Zhang Y. (2009). Genome scan of pigmentation traits in Friesian-Jersey crossbred cattle. J Genet Genom..

[44] Liang D., Zhao P., Si J., Fang L., Pairo-Castineira E., Hu X., Xu Q., Hou Y., Gong Y., Liang Z. (2021). Genomic Analysis Revealed a Convergent Evolution of LINE-1 in Coat Color: A Case Study in Water Buffaloes (*Bubalus bubalis*). Mol. Biol. Evol..

[45] Xu X., Dong G., Hu X., Miao L., Zhang X., Zhang D., Yang H., Zhang T., Zou Z., Zhang T. (2013). The genetic basis of white tigers. Curr. Biol..

[46] Caro T., Mallarino R. (2020). Coloration in Mammals. Trends Ecol. Evol..

[47] Goding C., Arnheiter H. (2019). MITF-the first 25 years. Genes Dev..

[48] Seberg H., Van O., Cornell R. (2017). Beyond MITF: Multiple transcription factors directly regulate the cellular phenotype in melanocytes and melanoma. Pigment Cell Melanoma Res..

[49] Gelmi M., Houtzagers L., Strub T., Krossa I., Jager M. (2022). MITF in Normal Melanocytes, Cutaneous and Uveal Melanoma: A Delicate Balance. Int. J. Mol. Sci..

[50] Ferguson J., Wilcock D., McEntegart S., Badrock A., Levesque M., Dummer R., Wellbrock C., Smith M. (2020). Osteoblasts contribute to a protective niche that supports melanoma cell proliferation and survival. Pigment Cell Melanoma Res..

[51] Hauswirth R., Haase B., Blatter M., Brooks S., Burger D., Drögemüller C., Gerber V., Henke D., Janda J., Jude R. (2012). Mutations in MITF and PAX3 cause “splashed white” and other white spotting phenotypes in horses. PLoS Genet..

[52] Johnston S., McEwan J., Pickering N., Kijas J., Beraldi D., Pilkington J., Pemberton J., Slate J. (2011). Genome-wide association mapping identifies the genetic basis of discrete and quantitative variation in sexual weaponry in a wild sheep population. Mol. Ecol..

[53] Shariflou M., Wade C., Kijas J., McCulloch R., Windsor P., Tammen I., Nicholas F. (2013). Brachygnathia, cardiomegaly and renal hypoplasia syndrome (BCRHS) in Merino sheep maps to a 1.1-megabase region on ovine chromosome OAR2. Anim. Genet..

[54] Zhou S., Zeng H., Huang J., Lei L., Tong X., Li S., Zhou Y., Guo H., Khan M., Luo L. (2021). Epigenetic regulation of melanogenesis. Ageing Res. Rev..

[55] Mo X., Kazmi H., Preston-Alp S., Zhou B., Zaidi M. (2022). Interferon-gamma induces melanogenesis via post-translational regulation of tyrosinase. Pigment Cell Melanoma Res..

[56] Ma S., Liu H., Wang J., Wang L., Xi Y., Liu Y., Xu Q., Hu J., Han C., Bai L. (2021). Transcriptome Analysis Reveals Genes Associated With Sexual Dichromatism of Head Feather Color in Mallard. Front Genet..

[57] Liu Q., Qi Y., Liang Q., Song J., Liu J., Li W., Shu Y., Tao M., Zhang C., Qin Q. (2019). Targeted disruption of tyrosinase causes melanin reduction in Carassius auratus cuvieri and its hybrid progeny. Sci. China Life Sci..

[58] Van L., Nguyen M., Braunschweig M., Nezer C., Collette C., Moreau L., Archibald A., Haley C., Buys N., Tally M. (2003). A regulatory mutation in IGF2 causes a major QTL effect on muscle growth in the pig. Nature.

[59] Hill M., Vande Z., Wittkopp P. (2021). Molecular and evolutionary processes generating variation in gene expression. Nat. Rev. Genet..

[60] Wang X., Zhong J., Gao Y., Ju Z., Huang J. (2014). A SNP in intron 8 of CD46 causes a novel transcript associated with mastitis in Holsteins. BMC Genom..

