# Supplementary material for: The Hoof Color of Australian White Sheep Is Associated with Genetic Variation of the MITF Gene
Source: Animals (Basel). 2023 Oct 15;13(20):3218. doi: 10.3390/ani13203218 (PMC10603658; doi:10.3390/ani13203218)
Supplement: Supplementary file 1 [file animals-13-03218-s001.zip › animals-2575341-supplementary.pdf]

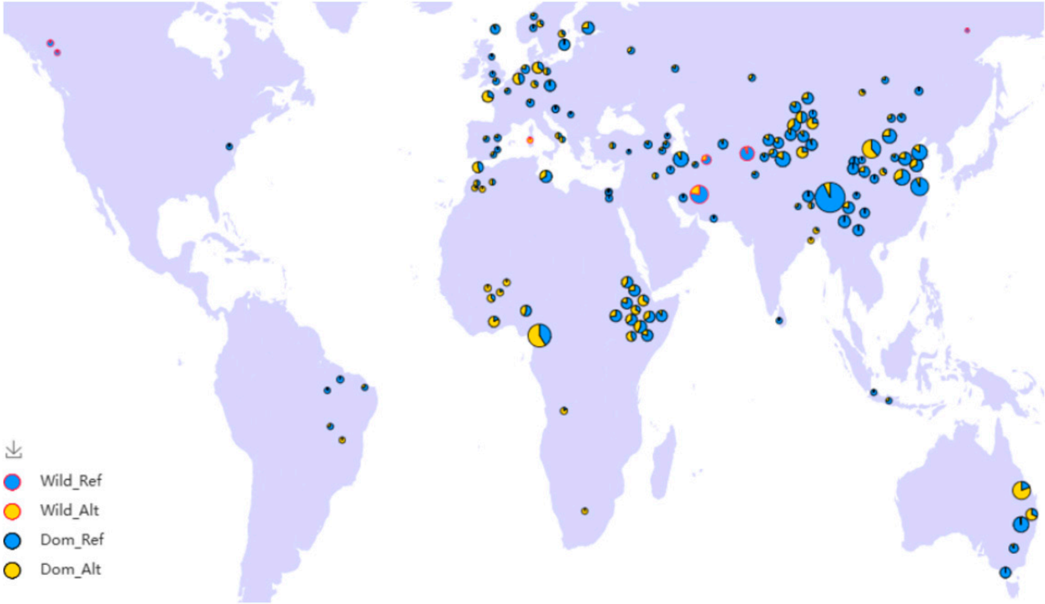

Supplementary Figure S1. Genotyping of the rs417454260 SNP in the global sheep population. These data were downloaded from the Ruminant Genome Database. Wild_Ref means wild sheep with the G allele; Wild_Alt means wild sheep with the A allele; Dom_Ref means domestic sheep with the G allele; Dom_Alt means domestic sheep with the A allele.

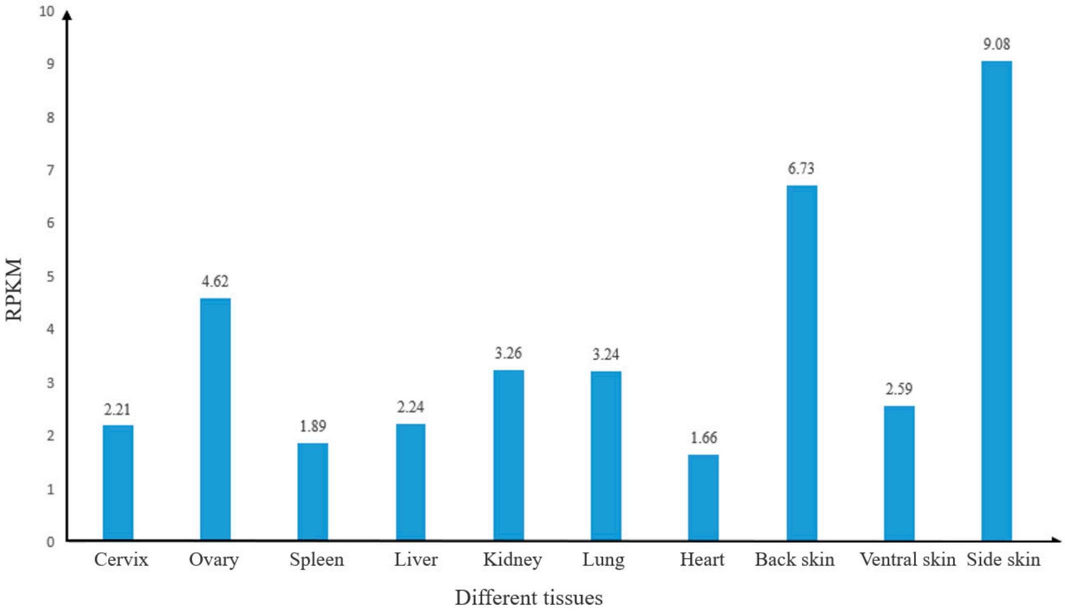

Supplementary Figure S2. The *MITF* gene expression in sheep tissues at adult stage. These data were downloaded from the Ruminant Genome Database.
